# Aflatoxin Decontamination in Maize Steep Liquor Obtained from Bioethanol Production Using Laccases from Species within the Basidiomycota Phylum

**DOI:** 10.3390/toxins16010027

**Published:** 2024-01-05

**Authors:** Marianela Bossa, María Silvina Alaniz-Zanon, Noelia Edith Monesterolo, María del Pilar Monge, Yamila Milagros Coria, Sofía Noemí Chulze, María Laura Chiotta

**Affiliations:** 1 Instituto de Investigación en Micología y Micotoxicología (IMICO), Consejo Nacional de Investigaciones Científicas y Técnicas (CONICET)—Universidad Nacional de Río Cuarto (UNRC), Ruta Nacional 36 Km 601, Río Cuarto 5800, Córdoba, Argentina; mbossa@exa.unrc.edu.ar (M.B.); malaniz@exa.unrc.edu.ar (M.S.A.-Z.); mmonge@exa.unrc.edu.ar (M.d.P.M.); yami.coria98@gmail.com (Y.M.C.); schulze@exa.unrc.edu.ar (S.N.C.); 2 Instituto de Biotecnología Ambiental y de la Salud (INBIAS), Consejo Nacional de Investigaciones Científicas y Técnicas (CONICET)—Universidad Nacional de Río Cuarto (UNRC), Ruta Nacional 36 Km 601, Río Cuarto 5800, Córdoba, Argentina; nmonesterolo@exa.unrc.edu.ar

**Keywords:** aflatoxins, maize, bioethanol co-products, laccases, decontamination

## Abstract

Maize (*Zea mays* L.) is an important crop in Argentina. *Aspergillus* section *Flavi* can infect this crop at the pre-harvest stage, and the harvested grains can be contaminated with aflatoxins (AFs). During the production of bioethanol from maize, AF levels can increase up to three times in the final co-products, known as, dry and wet distiller’s grain with solubles (DDGS and WDGS), intended for animal feed. Fungal enzymes like laccases can be a useful tool for reducing AF contamination in the co-products obtained from this process. The aim of the present study was to evaluate the ability of laccase enzymes included in enzymatic extracts (EE) produced by different species in the Basidiomycota phylum to reduce AF (AFB_1_ and AFB_2_) accumulation under the conditions of in vitro assays. Four laccase activities (5, 10, 15, and 20 U/mL) exerted by nine isolates were evaluated in the absence and presence of vanillic acid (VA), serving as a laccase redox mediator for the degradation of total AFs. The enzymatic stability in maize steep liquor (MSL) was confirmed after a 60 h incubation period. The most effective EE in terms of reducing AF content in the buffer was selected for an additional assay carried out under the same conditions using maize steep liquor obtained after the saccharification stage during the bioethanol production process. The highest degradation percentages were observed at 20 U/mL of laccase enzymatic activity and 1 mM of VA, corresponding to 26% for AFB_1_ and 26.6% for AFB_2._ The present study provides valuable data for the development of an efficient tool based on fungal laccases for preventing AF accumulation in the co-products of bioethanol produced from maize used for animal feed.

## 1. Introduction

Maize (*Zea mays* L.) is the most-produced cereal crop worldwide, as it is important to the economies of several countries, including Argentina. In Argentina, a relatively new application for maize is in bioethanol production, and the resulting by-products are intended for direct livestock use and feed production [1,2]. Conventional bioethanol is produced worldwide, mainly using maize or wheat [3]. In Argentina, bioethanol is produced from sugar cane and maize, with maize being the main source of this fuel. During 2022, the production of bioethanol from maize in Argentina reached 702,853 m^3^, while 455,988 m^3^ were obtained from sugar cane [4].

During bioethanol production, milling, liquefaction, enzymatic saccharification, fermentation, and ethanol purification are the main steps [5]. Milling can be wet or dry, with the second form being the most frequently employed to produce grits and flours, fats and fibre, and wet distillers grains with solubles (WDGS) or dried distillers grains with solubles (DDGS) [6,7,8,9,10]. At the end of the process, the ethanol is distilled, and solid residues are processed as sub-products to obtain nutritious animal feed [9].

The moisture content of WDGS is around 65%, and this sub-product is generally dried up to 10 to 12%, resulting in DDGS [11]. It is estimated that each tonne of dry maize grains yields 417 L of ethanol and 312 kg of DDGS [9,12]. These DDGS are preferred for animal feeding instead of WDGS since the lower moisture content confers uniform and better conditions during transportation and storage [13]. Furthermore, they have significant protein, amino acid, and phosphorus content [14].

The Food and Agriculture Organization of the United Nations (FAO) estimates that 25% of global crops can be infected by fungi and contaminated with different mycotoxins. This situation is precipitating economic losses worldwide [15]. Fungal infection and mycotoxin production are related to a crop’s susceptibility to infection by a specific fungal species and its eco-physiological behaviour in relation to water activity and temperature [16]. Both fungal infection and mycotoxin synthesis can occur at the pre-harvest stage, during the harvest, and at the post-harvest stage. Furthermore, an increase in mycotoxin levels during the handling and storage of grains may occur [17]. In this context, maize grains used in the bioethanol production process may contain high levels of mycotoxins and thus co-products like DDGS, constituting a risk for animal health.

The mycotoxin distribution in feed may be influenced, among other factors, by initial grain contamination as well as cereal processing [3,18]. During the production of bioethanol from maize, mycotoxins are practically not degraded and/or eliminated. Even more, several studies have demonstrated that mycotoxins are not present in the produced bioethanol but are three times more concentrated in DDGS [12,18,19,20,21,22]. Additionally, it has been shown that mycotoxins can affect the behaviour of the yeast used during fermentation, decreasing bioethanol yields [3,18]. The most relevant mycotoxins in relation to toxicity and food safety are mainly produced by fungal species of the genera *Aspergillus*, *Fusarium*, and *Penicillium*. *Aspergillus flavus*, belonging to *Aspergillus* section *Flavi*, is an aflatoxin (AF) type B producer; consequently, it belongs to the fungal species of the greatest concern for maize [23,24,25,26]. Among all AFs described, four types have been the most studied (AFB_1_, AFB_2_, AFG_1_, and AFG_2_), although the most important in terms of toxicity and occurrence is AFB_1_. This mycotoxin is the most toxic natural compound described, and it has been classified as a type 1 human carcinogen by the International Agency for Research on Cancer [27].

According to the prevalence of AFs in DDGS, Zhang et al. [28] analysed 182 DDGS samples from different bioethanol-producing industries in the USA, and around 6% of them were contaminated with AFB_1_ in levels ranging from 1.04 to 3.7 ng/g. In another study carried out on 52 samples from the USA and European countries, Oplatowska-Stachowiak et al. [29] detected up to 16 ng/g of AFB_1_ in 75% of the DDGS samples. Recently, contamination levels of around 1.47 ng/g of AFB_1_ were detected in 60 out of 186 DDGS samples in Brazil [21]. In general, AF contamination levels are relatively low. However, the occurrence of AFs varies every year according to the climatic conditions [30,31,32], and considering the current climate change scenario, regions where low incidence of AFs is detected nowadays may become hot spots for this contamination in few years [33,34,35,36,37]. This situation poses a risk for human and animal health in terms of food safety.

In this sense, it is important to monitor mycotoxin contamination levels and risks along the whole production chain. Therefore, different practices must be implemented before, during, and after harvest [38]. A large number of physical, chemical, and biological methods have been developed to mitigate mycotoxin contamination [39]. Among the biological strategies for reducing AFs at the pre-harvest stage, the efficacy of non-toxigenic *A. flavus* or *A. parasiticus* application in maize fields has been demonstrated in different countries, including the USA [40], Nigeria [41], Italia [42], Serbia [43], Ghana [44], and Argentina [45,46]. Many of these formulates are commercially available [47]. What is more, it has been demonstrated that the application of biocontrol agents at the field stage is an appropriate tool for reducing AFB_1_ accumulation during storage [46]. Although these strategies and their incorporation into an integrated pest management strategy are useful for preventing mycotoxin contamination, they cannot totally mitigate AFs. Consequently, it is necessary to complement these efforts during the processing of maize sub-products.

Several biological strategies for reducing AF levels at the post-harvest stage have great potential, such as the use of microorganisms or enzymes with the ability to metabolize, destroy, or transform toxins into less toxic, even innocuous, and stable compounds. Enzyme implementation in industrial processes is an efficient method for managing issues related to environmental sustainability, problems regarding the food and feed industries relating to activities such as baking and juice processing, and wastewater bioremediation [39,48,49]. For instance, the commercial product FUMzyme^®^ (Biomin, Tull, Austria) was evaluated at a pilot scale during the fermentation step in the production of bioethanol from maize, and an important reduction in the fumonisin contamination of DDGS was observed [50].

Laccase enzyme is a multi-copper (Cu)-containing polyphenol oxidase that was first detected in the exudates of *Rhusvernicifera*, the Japanese lacquer tree, in 1883 [49]. Nowadays, the synthesis of these enzymes has been demonstrated in plants, insects, bacteria, and fungus [51,52]. Fungal laccases have been identified among different species belonging to the phyla Ascomycota and Basidiomycota [53,54,55,56,57]. Among the fungal genera in Basidiomycota, those that cause white rot, such as *Pleurotus* and *Funalia* genera, stand out as abundant laccase producers [51]. The optimal conditions for laccases are an acidic pH and temperatures around 35–70 °C [48]. Their stability depends on several factors, such as the type of protein packaging, hydrophobicity, the number of intramolecular hydrogen bonds and salt bridges, the distribution of charged amino acid residues on the protein surface, and the content of certain amino acids [58].

Due to the redox potential of laccases that allows them to degrade a wide range of aromatic compounds, their role in the biotransformation of mycotoxins has recently been investigated [39,48,59,60,61,62,63]. Besides the great variety of compounds that laccases may oxidise, sometimes, synthetic or natural redox mediators may facilitate substrate oxidation by transferring electrons [48,64,65,66].

Based on the aforementioned background, the aim of the present study was to evaluate laccases produced by species within the Basidiomycota phylum and select a laccase able to reduce AF contamination with the potential for application in the bioethanol production process. This research contributes to the development of a tool based on fungal laccases that allows for the acquisition of innocuous maize sub-products intended for animal consumption.

## 2. Results

### 2.1. In Vitro Assays for Aflatoxin Degradation in Buffer Medium

**Figure 1:**
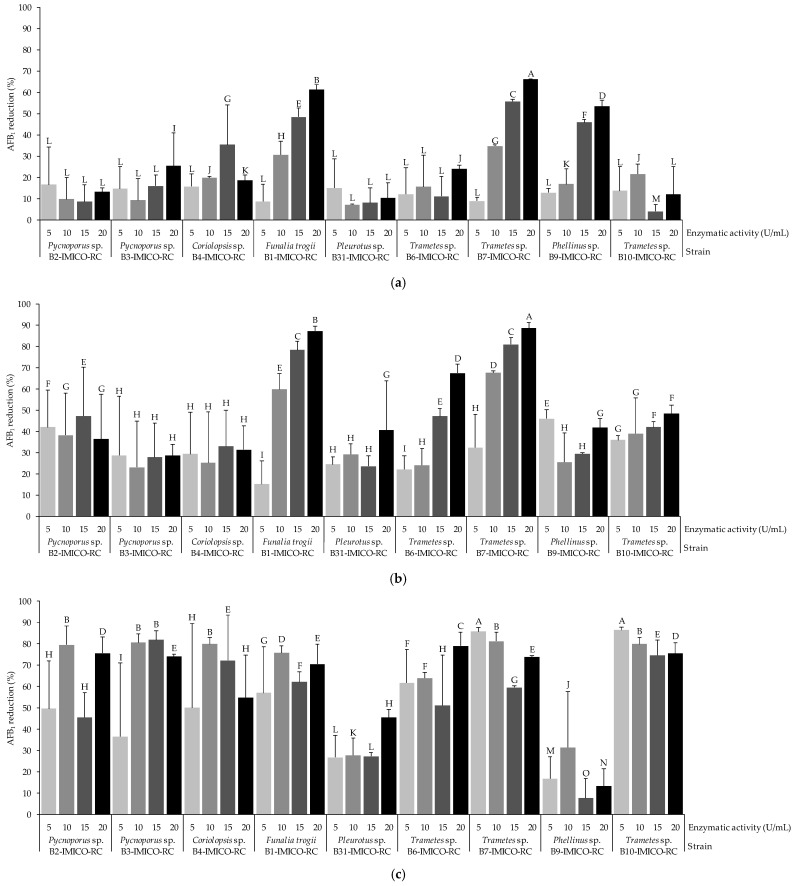
Reduction percentages of AFB_1_ under conditions of in vitro assays with buffer medium, treated with the enzymatic extracts containing laccases (5, 10, 15, and 20 U/mL) from different fungal strains in the absence (**a**) and presence of 1 mM (**b**) and 10 mM (**c**) of vanillic acid. A value of 100% represents the total reduction of AFB_1_ in comparison to the control (0% reduction). Error bars indicate the standard deviation of the replicates within each treatment. Different letters in each graphic indicate significant differences between treatments (*p* < 0.05).

Data on the ability of the enzymatic extracts (EEs) containing laccases to reduce aflatoxin B_1_ (AFB_1_) in the absence and presence of vanillic acid (VA), serving as a redox mediator, are shown in Figure 1. In the negative control, the AF levels remained stable throughout the 3-day incubation period. In the absence of VA, the highest reduction level (66.1%) was observed for the EE from the *Trametes* sp. strain B7-IMICO-RC at 20 U/mL of laccase activity (*p* < 0.05) (Figure 1a). The same EE at 15 U/mL was also effective in reducing AFB_1_ (55.7%). Moreover, the EE from *F. trogii* B1-IMICO-RC and *Phellinus* sp. B9-IMICO-RC significantly reduced AFB_1_ at 15 and 20 U/mL, reaching reduction percentages of 48.4 and 61.2% and 45.9 and 53.4%, respectively. Particularly, for these three EEs, the higher the laccase activity, the higher the AFB_1_ reduction percentages. The other EEs evaluated showed reduction percentages ranging from 4.1 to 35.5%.

In the presence of VA (1 mM), the AFB_1_ reduction percentages were higher than those observed when this redox mediator was not added (Figure 1b). The highest reduction percentages were detected for the EEs from the *Trametes* sp. B7-IMICO-RC (88.6%) and *F. trogii* B1-IMICO-RC (87.3%) strains at 20 U/mL of laccase activity (*p* < 0.05). Both EEs were also effective when evaluating the laccase activities at 10 and 15 U/mL, with reduction values ranging from 59.9 to 80.9% in comparison to the negative control. The EE from *Phellinus* sp. B9-IMICO-RC evaluated at activity levels of 15 and 20 U/mL showed less efficacy in reducing AFB_1_ in comparison with that exhibited in the assay in the absence of VA. Similar to the assay in which VA was absent, for the EEs from *Trametes* sp. B7-IMICO-RC and *Funalia trogii* B1-IMICO-RC, the higher the laccase activity, the higher the AFB_1_ reduction percentage. This behaviour was not observed for the EE from *Phellinus* sp. B9-IMICO-RC. The other EEs showed variable effectiveness depending on the laccase activity evaluated and the fungal strain from which the EE was obtained (22.2–67.6%).

When 10 mM of VA was added, the AFB_1_ reduction percentages were higher than those observed in the previous assays, with the exception of the EE from *Pleurotus* sp. B31-IMICO-RC and *Phellinus* sp. B9-IMICO-RC (Figure 1c). The EE from *Trametes* sp. B7-IMICO-RC as well as from *Funalia trogii* B1-IMICO-RC showed lower AFB_1_ reduction percentages at 15 and 20 U/mL of laccase activity in comparison with those observed in the assay with 1 mM of VA, whereas at 5 and 10 U/mL, these percentages increased (*p* < 0.05). The EE from *Trametes* sp. B7-IMICO-RC reduced AFB_1_ content by 85.7 and 81.2% when the laccase activities were 5 and 10 U/mL, respectively. At these laccase activity levels, the EE from *Funalia trogii* B1-IMICO-RC reduced AFB_1_ by 57.0 and 75.7%, respectively. In particular, the strain *Trametes* sp. B10-IMICO-RC induced significant AFB_1_ reduction at every laccase activity assayed (74.7–86.5%). The AFB_1_ reduction percentages induced by the EE from *Pleurotus* sp. B31-IMICO-RC did not vary when different concentrations of VA were used. In general, the EE from *Phellinus* sp. B9-IMICO-RC showed a weaker ability to reduce AFB_1_ levels when the VA concentration increased. As regards the other EEs, their efficacy in reducing AFB_1_ in the presence of VA (10 mM) depended on the laccase activity under study. In contrast with the previous experiments, the lowest laccase activities had similar effectiveness to the highest laccase activities (*p* < 0.05).

**Figure 2:**
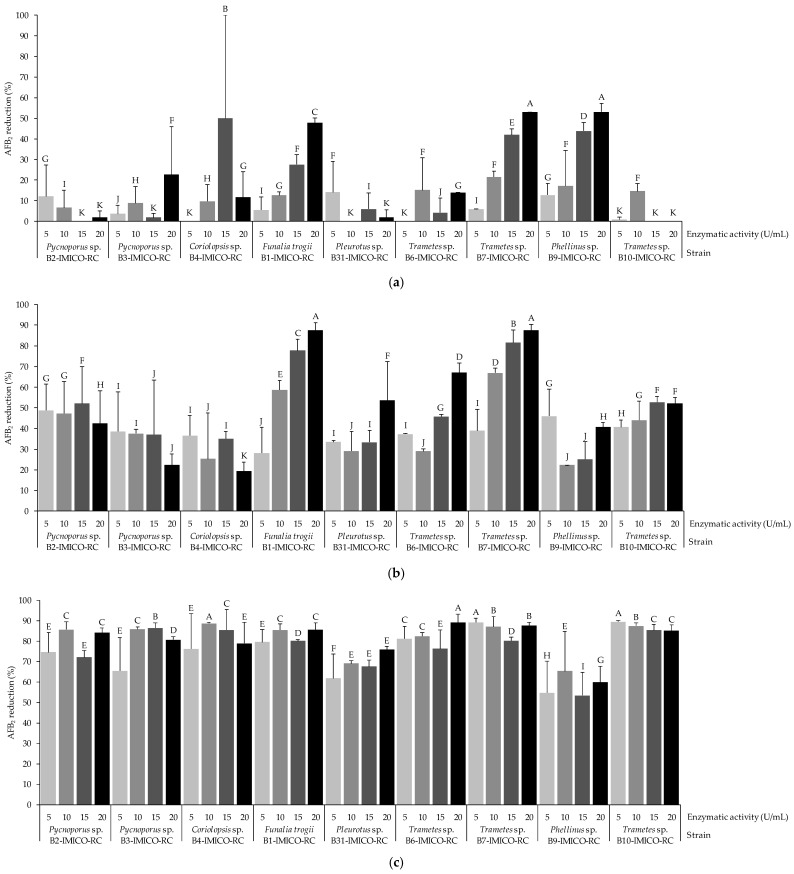
Reduction percentages of AFB_2_ under the conditions of the in vitro assays with buffer medium treated with the enzymatic extracts containing laccases (5, 10, 15, and 20 U/mL) from different fungal strains in the absence (**a**) and presence of 1 mM (**b**) and 10 mM (**c**) of vanillic acid. A value of 100% represents the total reduction of AFB_2_ in comparison to the control (0% reduction). Error bars indicate the standard deviation of the replicates within each treatment. Different letters in each graphic indicate significant differences between treatments (*p* < 0.05).

Regarding AFB_2_ reduction, the EE containing laccase obtained from *Trametes* sp. B7-IMICO-RC was one of the most effective in the absence of VA, mainly when the laccase activities were high (Figure 2a) (*p* < 0.05). The AFB_2_ reduction percentage achieved using this EE, as well as the EE from *Phellinus* sp. B9-IMICO-RC at 20 U/mL of laccase activity, was around 53%. When the VA concentration was increased, all the EEs evaluated showed higher reduction percentages (Figure 2b,c) in comparison with those observed in the assay performed in the absence of this redox mediator. The EE from *Trametes* sp. B7-IMICO-RC assayed at 1 mM of VA was the most effective at laccase activities of 10, 15, and 20 U/mL, reaching percentages of 66.9, 81.4, and 87.6%, respectively, whereas at 10 mM of VA, the highest AFB_2_ reduction (89.1%) induced by this EE was observed when the laccase activity was low (5 U/mL). Similar to the results for AFB_1_ reduction, the EE from *Trametes* sp. B7-IMICO-RC and *Funalia trogii* B1-IMICO-RC were more effective at reducing AFB_2_ accumulation when the laccase activity was higher in the absence of a redox mediator and with 1 mM of VA.

### 2.2. Laccase Stability in Maize Steep Liquor

In order to evaluate if maize steep liquor (MSL) could affect enzymatic activity throughout the measurement period, a laccase stability assay was carried out. Laccase activity was not detected in either the MSL sample or the negative control (MSL with sterile broth instead of EE containing laccase). The initial pH of the MSL was 5.7, and it did not vary until the end of the incubation period. However, in the negative control, a decrease in pH was detected (3.5), remaining within the range in which laccases are functional [67]. *Trametes* sp. B7-IMICO-RC, *Phellinus* sp. B9-IMICO-RC, and *Funalia trogii* B1-IMICO-RC were selected for this stability analysis since these strains showed the highest AFB_1_ reduction percentages under in vitro assays using a buffer without VA.

The EE from *Trametes* sp. B7-IMICO-RC showed a laccase stability of around 40% at the end of the incubation period. The EE from *Phellinus* sp. B9-IMICO-RC did not show laccase activity after 60 h of incubation. However, the laccase activity determined at 30 h of incubation showed a stability of around 15% in comparison with the beginning of the experiment. According to the EE from *Funalia trogii* B1-IMICO-RC, a stability of the laccase activity amounting to of 55% was observed. In agreement with the decrease in the laccase activity, the pH values also decreased at the end of the incubation period (by up to 3.5), as was observed for the negative control.

### 2.3. Aflatoxin Degradation in Maize Steep Liquor

**Figure 3:**
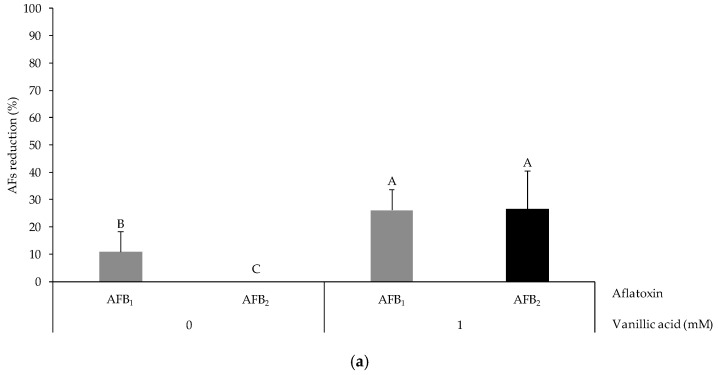

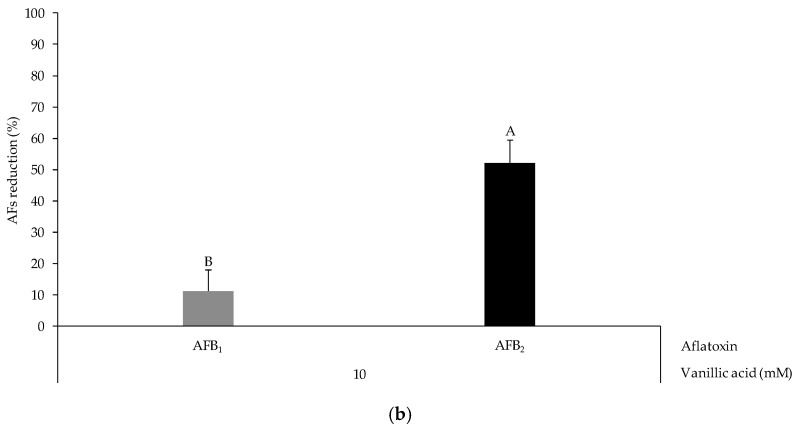
Reduction percentages of AFB_1_ and AFB_2_ under the conditions of in vitro assays in maize steep liquor treated with the enzymatic extracts containing laccases from *Trametes* sp. B7-IMICO-RC in different conditions: with 20 U/mL of laccase activity in the absence of vanillic acid and in the presence of 1 mM of vanillic acid (**a**) and with 5 U/mL of laccase activity in the presence of 10 mM of vanillic acid (**b**). A value of 100% represents the total reduction of AFs in comparison to the control (0% reduction). Error bars indicate the standard deviation of the replicates within each treatment. Different letters in each graphic indicate significant differences between treatments (*p* < 0.05).

The sample of MSL used for this assay was not naturally contaminated with either AFB_1_ or AFB_2_. Thus, additional amounts of AFs were added in order to carry out the experiment. For this assay, the EE from *Trametes* sp. B7-IMICO-RC was selected since it showed enzymatic stability under the simulated fermentation conditions and was effective in the AF degradation in buffer. The results showed that the EE containing laccases from *Trametes* sp. B7-IMICO-RC was able to reduce AFB_1_ levels in the MSL in the three experiments conducted (Figure 3). The optimal conditions for AFB_1_ reduction were 20 U/mL of laccase activity and 1 mM of VA, with the corresponding reduction percentages being 26.0 and 54.1%, respectively (*p* < 0.05). Aflatoxin B_2_ was also reduced by this EE only in the presence of VA, and the highest reduction level was detected at 10 mM of this redox mediator and 5 U/mL (a reduction of 52.0%). In general, the treatment that better reduced AFs in the MSL was the EE assayed at 20 U/mL of laccase activity and 1 mM of VA.

## 3. Discussion

Laccase enzymes are widely used as biotechnological tools since they are very versatile biocatalysts, and they are currently used in the food, textile, and pulp and paper industries as well as for other applications such as the detoxification of phenolic residual effluents [68]. Due to their potential to degrade a wide range of aromatic compounds and their successful application in the food industry, laccase enzymes have also been employed for the degradation of mycotoxins, such as aflatoxin B_1_ (AFB_1_) [59,60,69,70,71,72], AFG_1_, AFG_2_ and AFB_2_ [73], AFM_1_ [59], fumonisin B_1_ [60], zearalenone [60,63,74], and ochratoxin A [60]. However, the application of enzymes for mycotoxin detoxification in the food and feed industry is still limited [75]. In the present work, the degradation of total AFs by laccases contained in the enzymatic extracts (EEs) of various fungal strains belonging to the Phylum Basidiomycota was studied. The degradation ability of these mycotoxins was observed in a medium with a buffer and in a medium with maize steep liquor (MSL), either by the effect of a direct or indirect oxidation mediated by vanillic acid (VA) as a natural redox mediator.

This work remarks the relevance of evaluating EEs containing laccases from the nine evaluated strains, since all of them showed efficiency in degrading AFs. Additionally, these EEs reached similar or slightly lower AF reduction percentages compared to those previously reported by other authors, who analysed only one laccase with high potential for reducing AF content [59,60,63,66,69,71].

Several studies have shown that the use of redox mediators, which are low-molecular-weight molecules that are easily oxidised by laccases, favours mycotoxin degradation through the oxidation of the substrate outside the enzymatic active site [76]. To date, no results have been reported in relation to the use of VA as a mediator of mycotoxin oxidation via fungal laccase. However, the use of other compounds such as acetosyringone, ABTS, syringaldehyde, p-coumaric acid, ferulic acid, and TEMPO, among others, has been evaluated [59,60,66,72]. Acetosyringone and syringaldehyde, used as redox mediators, were the most effective at reducing AFB_1_, with percentages ranging between 68 and 90%. In studies using bacterial laccases, VA exhibited effectiveness in reducing AFB_1_, with reduction levels of 60 and 76% [62,63].

In the present study, the degradation levels of AFB_1_ induced by the EEs containing laccases were higher in the presence of the redox mediator than in the assay without VA. Additionally, in the absence and presence of low concentrations of VA, the highest laccase activities were needed for AFB_1_ degradation, whereas at higher VA concentrations, lower laccase activities were effective. Lloret et al. [77] concluded that the efficiency of the mediator depends on the affinity of the enzyme for the mediator, the stability of the oxidized intermediate compound, and the oxidation potential of the intermediate compound formed. They evaluated the effect of several natural and synthetic mediators in conjunction with laccase on the degradation of pharmaceutical products, demonstrating that at a lower concentration of mediator, there was no effect on the enzymatic activity, but with an increase, significant inactivation of the enzyme was observed. A good mediator should be a suitable substrate for an enzyme with a high turnover number, generate free radicals to disperse the active site for the oxidation of the final target compound, and yield an efficient result [78]. In the particular case of the EE containing laccases from *Phellinus* sp. B9-IMICO-RC strain, the addition of VA at any of the concentrations tested did not favour AFB_1_ reduction. This behaviour was independent of the laccase activity assayed. This was probably due to its lack of affinity for the mediator used. This is the first report about the ability of an EE containing laccases from a *Phellinus* sp. strain to reduce AFs. More studies on laccase produced by this genus should be carried out.

The EE containing laccases from the *Trametes* sp. B7-IMICO-RC strain was the most effective in reducing AFB_1_ in the absence of VA, although it showed higher reduction levels in the presence of this redox mediator. Previous studies have demonstrated the ability of laccases produced by the genera *Trametes* to degrade AFB_1_, achieving a reduction of 67–87% in the absence of redox mediators [69,71]. Although these reduction percentages are more promising than the results shown in the present study, the experimental conditions were different (purified laccases, enzymatic activity, and incubation time). The *F. trogii* strain also successfully degraded AFB_1_ under every condition assayed in the present study. Several white rot fungi species have been employed to degrade other toxic phenolic compounds, with *F. trogii* being one of the most studied species. Its particular relevance for the degradation of Bisphenol A and the decolorization of various dye solutions has been evidenced [79,80]. This effect is attributed to the release of different extracellular lignocellulosic enzymes, including laccases. In a study performed by Volobuev and Shakhova [81], strains of *Trametes versicolor* and *F. trogii* were recommended for biotechnological purposes since they combine two favourable characteristics: a high lignocellulolytic potential and a high growth rate.

As regards the degradation of AFB_2_, laccases contained in some of the evaluated EEs degraded up to 53.1 and 89.4% of this mycotoxin in the absence and presence of VA, respectively. Liu et al. [73] evaluated the degradation of AFs by a recombinant laccase from *Trametes* sp. expressed in *Saccharomyces cerevisiae* and observed a reduction percentage of 74%. This result is higher in comparison with the percentages reported in the present study in the absence of a redox mediator. Nevertheless, the addition of a low concentration of VA significantly improved the results, reaching 88% reduction. The molecular docking simulation developed by Liu et al. [73] predicted that among all the laccase–AF complexes evaluated, the lowest interaction was observed for laccase–AFB_2_, demonstrating a correlation with the lower reduction percentages under in vitro AFs degradation experiments in comparison with the other AFs evaluated. Moreover, other studies have demonstrated differences between the degradation rates of AFs type G and type B. Even when the laccase affinity for both AFB_1_ and AFG_2_ is similar for both AFs, AFG_1_ is more readily degraded due to its lack of side products and favourable binding dynamics [82]. More studies related to molecular docking using redox mediators would aid research regarding the prediction of AF biological degradation.

According to the enzymatic stability assay conducted in MSL, the laccases contained in the EEs from *Trametes* sp. B7-IMICO-RC and *F. trogii* B1-IMICO-RC remained active throughout the incubation period (60 h), whereas *Phellinus* sp. B9-IMICO-RC showed laccase activity at 30 h of incubation but not at 60 h. These results indicate that for most of the time and temperatures assayed, simulating the fermentation conditions employed at the bioethanol production process, the laccases remained active and had the potential to degrade AFs. It is relevant to know about the stability of laccase activity included in EEs after the fermentation stage during the bioethanol production process. If the laccases remained functional throughout the fermentation period, these enzymes would be able to decontaminate mycotoxins in MSL. The pH was adequate for laccase activity during the incubation time. The optimal pH for laccase greatly depends on the enzymatic substrate or culture medium used [51]. Without an effective and optimal pH, enzyme stability can be altered, subsequently making the enzymatic elimination of xenobiotics unachievable. Zeinvand-Lorestani et al. [71] showed that the optimal pH levels of *T. versicolor* laccase for AFB_1_ degradation were similar in the range detected in the evaluated MSL.

Aflatoxins were not detected in the MSL used in this assay, due to the detection limits of the methodology used. Thus, their levels did not exceed the limits established for total AFs by the Código Alimentario Argentino (CAA) or those regulated by the U.S. Food and Drug Administration (FDA) (in both cases, 20 ng/g) for maize and sub-products intended for human and animal consumption [83,84]. The CAA and the European Commission (EC) have established a limit of 5 ng/g for AFB_1_ in these substrates, and the sample did not surpass this value [83,85]. Nevertheless, some studies from different countries from around the world have reported the presence of mycotoxins in dried distillers grains with solubles (DDGS) in a range of 3–11 ng/g of AFB_1_ and up to 277 ng/g of total AFs [38,86,87]. It has also been confirmed that this concentration increases up to three times in DDGS when compared with levels detected in original maize grains [20]. Furthermore, the risk of the presence of AFs in maize will likely be higher as a result of global warming. Thus, the combination of both factors could favour greater AF contamination in DDGS. Postharvest decontaminating approaches such as the use of fungal laccases can be a last resource for solving issues regarding mycotoxins in food and feed.

In the AF degradation assay in MSL, the EE from *Trametes* sp. B7-IMICO-RC reduced AF contamination with different efficiencies depending on the evaluated conditions. The optimal conditions for AFB_1_ and AFB_2_ degradation were 20 U/mL of laccase activity and 1 mM of VA. This is the first report that includes an evaluation of the ability of an EE containing fungal laccases to degrade AFs in MSL obtained from the bioethanol production process. These results are comparable with those reported in the study by Loi et al. [88], who evaluated the decontamination of AFB_1_ in ground maize grains using purified laccase and observed a reduction of 25% in the presence of acetosyringone and dehydroascorbic acid as redox mediators.

When comparing AF degradation assays using the EE containing laccases of *Trametes* sp. B7-IMICO-RC, AF reduction was more efficient in the buffer than in MSL. In agreement with the present results, Loi et al. [60] observed reduction levels ranging from 11 to 73% in an in vitro assay using the same buffer media as that used in the present experiment. However, when they evaluated the same laccase applied to ground maize grains, the reduction percentages were lower (26%) [88]. These results show that when applying laccases to more complex systems such as ground maize grains or MSL, the efficiency of their enzymatic activity is reduced.

## 4. Conclusions

This is the first report evaluating the ability of fungal laccases to degrade aflatoxins (AFs) using maize steep liquor (MSL) obtained from the step prior to the fermentation during the bioethanol production process.

In summary, the enzymatic extract (EE) containing laccase from *Trametes* sp. B7-IMICO-RC applied in the buffer medium and in MSLwas effective in reducing AFB_1_ and AFB_2_ accumulation both in the absence of a redox mediator and in combination with vanillic acid (VA), with the latter being used as a natural and economical compound. The results from the in vitro assay conducted in the buffer could be useful for the development of “green tools” for application in the food and feed industries. Moreover, the results from the AF degradation analysis conducted in MSL demonstrate that the application of laccase enzymes, which are stable in this matrix, can decrease AF accumulation in bioethanol co-products obtained from maize.

Considering climate change and the consequent risk of mycotoxin accumulation, as well as the global need to develop new eco-friendly technologies, the application of fungal laccases is a promising tool. In the current scenario of sustainability, the implementation of biological agents and/or their metabolites, such as enzymes, into industrial processes is a specific and efficient strategy with a low impact on feed and food quality. The strategy proposed in the present work could reduce the risk of the accumulation of AFs in maize sub-products obtained during the bioethanol production process and in other food and feed products, contributing to food safety and adding value to the maize agri-food chain for the transformation of grains into by-products destined for animal feeding.

## 5. Materials and Methods

### 5.1. Strains

The fungal strains included in the present work were *Funalia trogii* B1-IMICO-RC, *Pleurotus* sp. B31-IMICO-RC, *Pycnoporus* sp. B2-IMICO-RC, *Pycnoporus* sp. B3-IMICO-RC, *Coriolopsis* sp. B4-IMICO-RC, *Trametes* sp. B6-IMICO-RC, *Trametes* sp. B7-IMICO-RC, *Phellinus* sp. B9-IMICO-RC, and *Trametes* sp. B10-IMICO-RC strains belonging to Phylum Basidiomycota. The strains were maintained in 20% glycerol at −80 °C in the culture collection of the Instituto de Investigación en Micología y Micotoxicología (IMICO), CONICET-UNRC, Argentina.

### 5.2. Production of Enzymatic Extracts Containing Laccases

For enzymatic extract (EE) production, two plugs of mycelium developed on Potato Dextrose Agar (PDA) plates were transferred to 250 mL flasks with 50 mL of sterile broth containing malt extract (2% *w/v*), yeast extract (5% *w/v*), 0.1 mM of CuSO_4_, and 100 mL of 10 mM potassium phosphate buffer (pH 6) [59]. The cultures were incubated for 24 days in the dark at 30 ± 1 °C under static conditions. After this period, the fungal mycelium was removed via filtration through 0.22 µm nylon syringe filters (Titan^®^). The EEs were used for laccase activity determination. Laccase activity was determined via the oxidation of 2,2′-azino-di-3-ethylbenzothiazoline (ABTS) (Sigma-Aldrich, St. Louis, MO, USA) (ε_420_ = 36,000/M·cm), serving as the enzyme substrate, at a wavelength of 420 nm using a spectrophotometer (UV-VISIBLE Rayleigh LUV200B). The reaction was carried out according to the process reported by Montoya et al. [89] with some modifications. For each reaction, 0.1 M sodium acetate buffer (pH 3.6), 0.5 mM of ABTS, and an appropriate amount of the EE were combined to obtain a final volume of 1 mL. One unit (U) of enzyme activity was defined as the enzyme activity required to oxidise 1 μmol of ABTS per minute at 25 ± 1 °C.

### 5.3. In Vitro Assays for Aflatoxin Degradation in Buffer Medium

Degradation of AFs by the EEs containing laccases was evaluated according to the methodology proposed by Loi et al. [60] with some modifications. Different working solutions of the EEs from each strain were prepared in 1 mM sodium acetate buffer (pH 5) to obtain final laccase activities of 5, 10, 15, and 20 U/mL. Independently, working solutions of AFs were prepared in acetonitrile to reach final concentrations of 1000 ng/mL of AFB_1_ and 250 ng/mL of AFB_2_. Finally, sodium acetate buffer (pH 5, reaction buffer) was added to obtain a final volume of 500 µL (1 mM). In the negative control for laccase activity, the EE was replaced by an equal volume of the sterile broth, which was previously described (Section 5.2.). This assay was performed with three different concentrations of vanillic acid (VA) as redox mediator, namely, 0 (absence), 1, and 10 mM, independently. The reactions were incubated at 30 ± 1 °C for 3 days in the dark. All reactions and control tubes were performed in triplicate, respectively. The reactions were stopped in boiling water for 5 min [74].

The AF analysis was carried out via HPLC according to the methodology described by Alaniz Zanon et al. [46]. Briefly, 200 μL of each degradation reaction was derivatised by adding 700 μL of a solution of trifluoroacetic acid/glacial acetic acid/water (20:10:70, *v*/*v*/*v*) at a temperature of 65 ± 1 °C for 8.5 min. The detection and quantification of AFs was carried out by injecting 20 μL of each sample into a Waters Alliance e2695 HPLC system (Waters Corporation, Milford, MA, USA) coupled with a fluorescence detector (Waters 2998). Chromatographic separation was performed on a stainless steel C_18_ reversed phase column (150 mm × 4.6 mm i.d., particle size 5 µm; Luna-Phenomenex, Torrance, CA, USA) connected to a C_18_ pre-column (20 mm × 4.6 mm i.d., particle size 5 µm, LunaPhenomenex, Torrance, CA, USA). The mobile phase was water/methanol/acetonitrile (4:1:1, *v*/*v*/*v*), and the flow rate was 1.5 mL/min. The detection limit was 1 ng/mL for AFB_1_ and 0.8 ng/mL for AFB_2_. Pure AF solutions were used as external standards (Sigma-Aldrich, St. Louis, MO, USA).

### 5.4. Laccase Stability Evaluation in Enzymatic Extracts Applied to Maize Steep Liquor

For this analysis, MSL was collected prior to the application of *Saccharomyces cerevisiae* from a bioethanol production industry. The sample (5 L) was transferred to the laboratory to determine both pH (Model 250A, ORION) and AF natural incidence. The three most efficient strains in degrading AFB_1_ under the conditions of the in vitro assay carried out in the buffer were selected. The EEs from these strains were added to the MSL to obtain a final laccase activity of 20 U/mL. The reaction mixture consisted of 4 mL of the EE and 10 mL of the MSL, representing 95% of the Falcon tube capacity, resembling the occupation of the fermentation tank. In the negative control, the EE was replaced by the same volume of sterile broth. Control and treatment reactions were carried out in triplicate, and the samples were incubated at 33 ± 1 °C during 60 h under static conditions in the dark. These conditions of temperature and incubation time were selected since they are used in the fermentation step during the bioethanol production process. The samples were centrifuged at 10,000 rpm for 5 min (Sorvall ST 16, Thermo Fisher Scientific, Waltham, MA, USA). The supernatant was collected to determine pH and laccase activity (described in Section 5.2) at 0, 30, and 60 h.

### 5.5. In Vitro Assay for Aflatoxin Degradation in Maize Steep Liquor

#### 5.5.1. Aflatoxin Natural Incidence in Maize Steep Liquor Samples

The MSL sample used for the evaluation of AF degradation was the same as that used for the stability assay. Aflatoxin natural incidence in MSL was determined before the degradation assay. For this analysis, the sample was dried using a TDSF/A60 forced air oven (Tecno Dalvo, Santa Fe, Argentina) at 40 ± 1 °C until dryness was reached. The dried MSL was finely ground with a Romer propeller grinder (Romer Labs Inc., Union, MO, USA). Aflatoxin extraction was performed using a Quick, Easy, Cheap, Effective, Rugged, and Safe extraction procedure (QuEChERS), according to the methodology proposed by Bursić et al. [90] with some modifications. From the conditioned and homogenised MSL sample, 2.5 g was mixed with 7.5 mL of a mixture composed of 100 mL of 1% acetic acid in acetonitrile and 50 mL of water and shaken vigorously for 1 min. Subsequently, 0.45 g of anhydrous sodium acetate was added and immediately shaken for 2 min. Anhydrous magnesium sulphate (1.15 g) was added and vigorously stirred. Finally, the mixture was centrifuged for 20 min at 2500 rpm (Macro 80-2B, HudTech-Lancet) and stored overnight at −20 ± 1 °C. Four millilitres of the supernatant was recovered and evaporated until dryness was achieved. Aflatoxins were detected and quantified via HPLC according to the methodology described by Alaniz Zanon et al. [46]. The AF extraction was carried out in triplicate. For the calibration curve, a reference AF standard was used (Sigma-Aldrich, St. Louis, MO, USA). The limit of detection was 1 ng/g for AFB_1_ and 0.8 ng/g for AFB_2_.

#### 5.5.2. Aflatoxin Degradation Assay in Maize Steep Liquor

The EEs containing laccases from the strain that showed stability in MSL and a high percentage of AF degradation in the in vitro assay using buffer was selected for this experiment. The EE was evaluated at laccase activities of 5 U/mL with VA (10 mM) and 20 U/mL in the absence and presence of VA (1 mM). These conditions were the most effective for reducing AFs in the buffer via the selected EE. The total AFs were added to each reaction mixture to obtain final concentrations of 80 ng/g of AFB_1_ and 20 ng/g of AFB_2_. The negative control was developed replacing the volume of EE with sterile broth. The assay was carried out at 33 ± 1 °C over 60 h with an occupancy percentage of 95% (the same parameters used during the fermentation stage). All reactions were performed in triplicate. Finally, the reactions were stopped via placement in a boiling water bath for 5 min to enable laccase inactivation. The remaining AFs were detected as described in Section 5.5.1.

### 5.6. Statistical Analysis

Data on AF degradation percentages in buffer and in MSL were subjected to nonparametric Kruskal–Wallis test followed by Bonferroni’s post hoc comparison method at a probability level of *p* < 0.05. The statistical analysis were performed using InfoStat version 2020p software [91].

## Data Availability

Data is contained within the article.
